# Association between adherence to the Japanese diet and all-cause and cause-specific mortality: the Japan Public Health Center-based Prospective Study

**DOI:** 10.1007/s00394-020-02330-0

**Published:** 2020-07-16

**Authors:** Sanae Matsuyama, Norie Sawada, Yasutake Tomata, Shu Zhang, Atsushi Goto, Taiki Yamaji, Motoki Iwasaki, Manami Inoue, Ichiro Tsuji, Shoichiro Tsugane, the Japan Public Health Center-based Prospective Study Group

**Affiliations:** 1 Division of Epidemiology, Department of Health Informatics and Public Health, School of Public Health, Tohoku University Graduate School of Medicine, 2-1, Seiryo-machi, Aoba-ku, Sendai, Miyagi 980-8575 Japan; 2 Epidemiology and Prevention Group, Center for Public Health Sciences, National Cancer Center, Tokyo, Japan

**Keywords:** Japanese diet, Dietary pattern, Mortality, Prospective study

## Abstract

**Purpose:**

The present study aimed to examine the association between adherence to the Japanese diet and the subsequent risk of all-cause and cause-specific mortality using a large-scale cohort from settings all over Japan.

**Methods:**

We analyzed data from a cohort study of 92,969 Japanese adults aged 45–74 years, covering 11 public health center areas nationwide. We collected dietary information using a validated 147-item food frequency questionnaire. Adherence to the Japanese diet consisting of eight components (high intake of rice, miso soup, seaweeds, pickles, green and yellow vegetables, fish, and green tea; low intake of beef and pork) was assessed using 8-item Japanese Diet Index (JDI8) score, with scores ranging from 0 to 8. The Cox proportional hazards model was used to estimate the hazard ratio (HR) and 95% confidence interval (CI) for all-cause and cause-specific mortality.

**Results:**

During a median follow-up of 18.9 years, we documented 20,596 deaths. A higher JDI8 score was significantly associated with a lower risk for all-cause and cardiovascular disease (CVD) mortality. The multivariable-adjusted HR of all-cause and CVD mortality for the highest JDI8 score group (score of 6–8) versus the lowest JDI8 score group (score of 0–2) were 0.86 (95% CI 0.81–0.90, *P* trend < 0.001), and 0.89 (95% CI 0.80–0.99, *P* trend = 0.007), respectively.

**Conclusions:**

Adhering to the Japanese diet, as assessed by the JDI8, was associated with a decreased risk of all-cause and CVD mortality among adults living in multiple areas across Japan.

**Electronic supplementary material:**

The online version of this article (10.1007/s00394-020-02330-0) contains supplementary material, which is available to authorized users.

## Introduction

Japan has the world’s longest life expectancy (81.1 years for men and 87.1 years for women) [1]. This may be partly attributable to the Japanese dietary pattern. In fact, it has been reported that the Japanese dietary pattern was associated with a lower risk for all-cause and cardiovascular disease (CVD) mortality [2, 3]. However, evidence on the association between the Japanese diet and mortality risk is still lacking.

The 8-item Japanese Diet Index (JDI8) has been used to assess the degree of adherence to the Japanese diet [2, 4]. The JDI8 consists of the following eight components: high intake of rice, miso soup, seaweeds, pickles, green and yellow vegetables, fish, and green tea; and low intake of beef and pork. Studies have shown that a higher JDI8 score is associated with health benefits such as a lower risk of mortality [2] and disability [4]. However, these previous studies were conducted in a specific rural area of Japan. The Japan Public Health Center-based Prospective (JPHC) Study conducted a baseline survey of 140,420 registered residents aged 40–69 years within 11 public health center areas nationwide from 1990–1994 [5].

The aim of the present study was to examine the association between adherence to the Japanese diet assessed by JDI8 and the subsequent risk for all-cause and cause-specific mortality using data from a large-scale cohort from settings all over Japan.

## Materials and methods

### Study cohort

The JPHC Study was launched in 1990 and 1993 for cohorts I and II, respectively [5]. Participants in cohort I included residents aged 40–59 years from five Japanese public health center areas (Iwate, Akita, Nagano, Okinawa-Chubu, and Tokyo), and those in cohort II included residents aged 40–69 years from six other Japanese public health center areas (Ibaraki, Niigata, Kochi, Nagasaki, Okinawa-Miyako, and Osaka). A baseline survey questionnaire was distributed to 140,420 registered residents mostly by hand. Participants were informed of the objectives of the study, and that completion of the survey questionnaire was regarded as providing consent to participate. The 5- and 10-year follow-up surveys (second and third surveys, respectively) were conducted to update information on lifestyle habits and health conditions in 1995–1998 and 2000–2003, respectively. The present study used the second survey as the baseline.

**Fig. 1:**
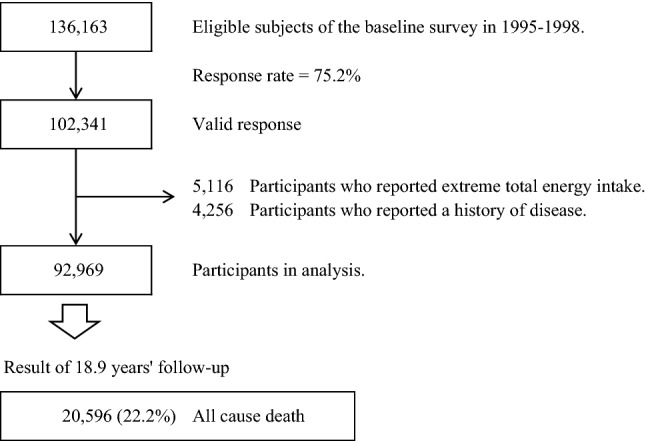
Flowchart of study participants

A total of 136,163 subjects were eligible for participation in this study. Of those, 102,341 participants (75.2%) who provided valid responses formed the study cohort. We excluded 5116 participants who reported extreme total energy intake (sex-specific values outside of ± 2.5%) and 4256 participants who reported a history of disease, including cancer, stroke, myocardial infarction, and chronic liver disease. Thus 92,969 participants (42,700 men and 50,269 women) were analyzed (Fig. 1).

#### *Exposure* (8-item Japanese Diet Index score)

A semi-quantitative food frequency questionnaire (FFQ) was used to assess the average intake of 147 food and beverage items in the second survey [6, 7]. For most food items, participants were asked about consumption frequency and usual portion size. The validity and reproducibility of the FFQ have been established in previous studies [8–10].

Based on previous studies [2, 4], we used the JDI8 to assess the degree of adherence to the Japanese diet. The JDI8 consists of eight components: rice, miso soup, seaweeds, pickles, green and yellow vegetables (green vegetables, carrot, pumpkin, and tomato), fish (raw fish, salty fish, dried fish, seafood, canned tuna, and fish products), green tea, and beef and pork (beef, pork, and processed meat). The first seven components represent adherence to the Japanese diet, and participants received one point if their intake was more than or equal to the sex-specific median. The 8th component represents non-adherence to the Japanese diet, and participants received one point if their intake was less than the sex-specific median. Supplemental Table 1 shows the sex-specific median of intake of the JDI8 components. The JDI8 score ranged from 0 to 8, with higher scores indicating greater dietary conformity. Incidentally, the JDI8 was modified to exclude coffee from the original JDI, because lower coffee consumption was significantly associated with an increased risk of all-cause mortality [2]. Additionally, previous studies using a meta-analysis approach reported that coffee consumption was inversely associated with all-cause and CVD mortality [11].

### Endpoints

The participants’ residency and vital status were followed up from baseline to December 31, 2016, using the residential registry. Causes of death were confirmed by death certificates and were defined according to International Classification of Diseases, 10th revision (ICD-10). The major endpoints of this study were mortality from all-cause, cancer (ICD-10: C00–C97) and CVD (ICD-10: I00–I99). CVD mortality was subdivided into mortality from heart disease (ICD-10: I20–I52) and cerebrovascular disease (ICD-10: I60–I69).

### Ethical issues

This study was approved by the Institutional Review Board of the National Cancer Center of Japan (approval code; 2015-085) and the Ethics Committee of Tohoku University Graduate School of Medicine (approval code; 2018-1-321).

### Statistical analysis

We counted the person-years of follow-up for each subject from the date of response to the second survey questionnaire until the date of death or the end of the study period (December 31, 2016), whichever occurred first.

The adjusted Cox proportional hazards model was used to calculate the hazard ratios (HRs) and 95% confidence intervals (CIs) for all-cause and cause-specific mortality according to the groups of the JDI8 score (G1, score of 0–2; G2, score of 3; G3, score of 4–5; and G4, score of 6–8). The lowest group was used as the reference category. Multivariable-adjusted models were adjusted for the following variables. Model 1 was adjusted for age (45–49, 50–54, 55–59, 60–64, 65–69, or ≥ 70 years), sex, and study area (11 areas). To examine whether the association between adherence to the Japanese diet and mortality was attributable to physical health status or other lifestyle factors, Model 2 was further adjusted for body mass index (< 18.5, 18.5–24.9, 25–29.9, ≥ 30 kg/m^2^, or missing), smoking status (current, former, never, or missing), alcohol drinking (< 1 time/month, 1–3 times/month, 1–2, 3–4, 5–6 times/week, every day, or missing), total physical activity (quartile of metabolic equivalent task-hours/day), medication (antihypertensive, cholesterol-lowering, or hypoglycemic agents [yes or no for each item]), and occupation (agriculture, forestry, fishery, office work, self-employed, specialty work, housewife, unemployed, or other [yes or no for each item]). In addition to these adjustments, Model 3 was further adjusted for total energy intake (in kcal/d; sex-specific quartile categories). To test for linear trends, categories indicating an increase in the JDI8 score (scored as 1 for G1, 2 for G2, 3 for G3, and 4 for G4), and variables using the JDI8 score as a continuous variable were entered in the corresponding Cox model. The proportional hazards assumption was checked based on the Schoenfeld residual test using Stata command "estat phtest". In this analysis, the proportional hazards assumption was not rejected with the Schoenfeld residual test (*P* = 0.275).

We also conducted several sensitivity analyses to test the robustness of our findings. First, considering possible reverse causality, we conducted an analysis after exclusion of deaths occurring in the first 3 years of follow-up (*n* = 91,589). Second, we conducted analyses stratified by sex to assess whether effects of adherence to the Japanese diet on mortality varied by sex (male = 42,700, female = 50,269). Third, to minimize potential bias attributed to total energy intake, we calculated the JDI8 score using the data after adjustment by the residual method (*n* = 92,969). Fourth, for the same reason (i.e., to minimize potential bias attributed to total energy intake), we also analyzed the association including participants who reported extreme total energy intake (*n* = 97,802). Fifth, we performed an analysis that included sodium and soybean products (tofu, fermented soybean [natto], and soymilk, etc.) intakes in the covariates because it was reported that the Japanese diet was associated with high sodium intake [12, 13], and soybean products are characteristic of the Japanese diet [14] (*n* = 92,969). In addition, we investigated the relationship between each component of the JDI8 and all-cause and cause-specific mortality. Here, we conducted an analysis for each item by including it into the Cox model with full adjustment, respectively.

The Cox proportional hazards model was performed using SAS version 9.4 (SAS Inc., Cary, NC, USA). The proportional hazards assumption was performed by Stata/MP15 (StataCorp, Texas, USA). All statistical tests described here were two-sided, and differences at *P* < 0.05 were considered significant.

## Results

Among the 92,969 participants, the proportion of men was 45.9%, the mean age (standard deviation [SD]) was 56.5 (7.8) years, median follow-up time was 18.9 years, follow-up rate was 99.7%, and follow-up for cause of death (in decedents) was over 98.0%.

Table 1 shows characteristics of study participants according to the JDI8 score groups. Participants with a higher JDI8 score were older, more likely to be male, to drink alcohol every day, to engage in more physical activity, to have greater total energy intake, and less likely to have high BMI (25.0 to < 30.0 and ≥ 30 kg/m^2^). In contrast, participants with a lower JDI8 score were younger and current smokers.

**Table 1:** Characteristics according to groups of the JDI8 score (*n* = 92,969)

Characteristic	Groups of the JDI8 score			
	G1 (low)	G2	G3	G4 (high)
The JDI8 score	0–2	3	4, 5	6–8
No. of participants	16,838	15,461	36,196	24,474
Age (years), mean (SD)	54.6 (7.9)	56.0 (8.0)	57.0 (7.9)	57.4 (7.3)
Males, *n* (%)	7302 (43.4)	6987 (45.2)	16,476 (45.5)	11,935 (48.8)
Body mass index (kg/m^2^), *n* (%)				
< 18.5	640 (3.9)	622 (4.1)	1345 (3.8)	835 (3.5)
18.5–25.0	10,810 (65.5)	10,078 (66.5)	24,170 (67.9)	16,802 (69.3)
25.0–30.0	4348 (26.3)	3955 (26.1)	8959 (25.2)	5915 (24.4)
≥ 30.0	706 (4.3)	509 (3.3)	1125 (3.1)	680 (2.8)
Current smoker, *n* (%)	4,151 (26.2)	3,618 (24.9)	8,187 (24.1)	5,698 (24.5)
Drink alcohol every day, *n* (%)	3,096 (18.8)	2,851 (18.9)	7,137 (20.2)	5,446 (22.6)
Total physical activity (MET-h/day), mean (SD)	31.0 (6.2)	31.3 (6.4)	32.0 (6.5)	32.9 (6.6)
Medication, *n* (%)				
Antihypertensive	2792 (16.6)	2935 (19.0)	7155 (19.8)	4743 (19.4)
Cholesterol-lowering	702 (4.2)	789 (5.1)	2123 (5.9)	1499 (6.1)
Hypoglycemic agents	441 (2.6)	489 (3.2)	1112 (3.1)	661 (2.7)
Occupation, *n* (%)				
Agriculture	1898 (11.3)	2578 (16.7)	8164 (22.6)	7466 (30.5)
Forestry	28 (0.2)	70 (0.5)	192 (0.5)	190 (0.8)
Fishery	200 (1.2)	252 (1.6)	693 (1.9)	337 (1.4)
Office work	5360 (31.8)	4479 (29.0)	9948 (27.5)	6636 (27.1)
Self-employed	2496 (14.8)	2144 (13.9)	5107 (14.1)	3507 (14.3)
Specialty work	1334 (7.9)	1058 (6.8)	2159 (6.0)	1436 (5.9)
Housewife	3695 (21.9)	3400 (22.0)	8413 (23.2)	5762 (23.5)
Unemployed	1403 (8.3)	1523 (9.9)	3427 (9.5)	2107 (8.6)
Other	1572 (9.3)	1286 (8.3)	2732 (7.6)	1658 (6.8)
Total energy intake (kcal/day), mean (SD)	1656 (506)	1782 (543)	2005 (582)	2337 (598)
Sodium intake (mg/day), mean (SD)	3173 (1,348)	3920 (7,298)	4901 (1,971)	6466 (2,299)
Potassium intake (mg/day), mean (SD)	1933 (744)	2272 (907)	2840 (1,061)	3668 (1,186)
Sodium–potassium ratio, mean (SD)	1.7 (0.6)	1.8 (1.7)	1.8 (0.5)	1.8 (0.5)
Components of Index score + 1 point given, *n* (%)				
Adhering components^†^				
Rice	4,483 (26.6)	7,531 (48.7)	22,499 (62.2)	20,471 (83.6)
Miso soup	3,690 (21.9)	7,176 (46.4)	22,682 (62.7)	20,675 (84.5)
Seaweeds	2,142 (12.7)	4,702 (30.4)	19,490 (53.9)	20,578 (84.1)
Pickles	1,392 (8.3)	3,686 (23.8)	19,703 (54.4)	21,704 (88.7)
Green and yellow vegetables	2,471 (14.7)	4,759 (30.8)	18,590 (51.4)	20,665 (84.4)
Fish	2,183 (13.0)	4,464 (28.9)	19,377 (53.5)	20,462 (83.6)
Green tea	3,436 (20.4)	6,289 (40.7)	21,698 (60.0)	20,148 (82.3)
Non-adhering components^‡^				
Beef and pork	6,148 (36.5)	7,776 (50.3)	18,718 (51.7)	13,849 (56.6)

^†^Participants received + 1 point if their intake was more than or equal to the sex-specific median

^‡^Participants received + 1 point if their intake was below the sex-specific median

During 1,635,302 person-years of follow-up, we identified 20,596 all-cause deaths, 7148 cancer deaths, 4990 CVD deaths, including 2600 heart disease deaths and 1950 cerebrovascular disease deaths. Table 2 shows the association between groups by JDI8 score and all-cause, cancer, CVD, heart disease, and cerebrovascular disease mortality, along with HRs and 95% CIs. The JDI8 score was inversely associated with risk of all-cause, CVD, and heart disease mortality. The multivariable-adjusted HR (95% CI) of all-cause, CVD, and heart disease mortality for the highest group of the JDI8 score was 0.86 (0.81–0.90, *P* trend < 0.001), 0.89 (0.80–0.99, *P* trend = 0.007) and 0.89 (0.77–1.03, *P* trend = 0.037), respectively compared to the lowest group. In addition, each one-point increase in the JDI8 score was linearly and inversely associated with mortality risk. The multivariable-adjusted HR (95% CI) of all-cause, CVD, and heart disease mortality for each one-point increase of the JDI8 score was 0.97 (0.96–0.98, *P* trend < 0.001), 0.97 (0.95–0.99, *P* trend = 0.003) and 0.96 (0.94–0.99, *P* trend = 0.008), respectively. The risk of cancer and cerebrovascular disease mortality decreased with higher JDI8 score after adjustment for age, sex, and area. However, these associations were attenuated after adjustment for covariates. The multivariable-adjusted HR (95% CI) of cancer and cerebrovascular disease mortality for the highest JDI8 score group was 0.94 (0.86–1.03, *P* trend = 0.175) and 0.89 (0.75–1.05, *P* trend = 0.237), respectively compared to the lowest JDI8 score group.

**Table 2:** Relationship between the JDI8 score and mortality (*n* = 92,969)

	Groups of the JDI8 score				Each one-point increase of the JDI8 score	*P* trend
	G1 (low)	G2	G3	G4 (high)		
	(*n* = 16,838)	(*n* = 15,461)	(*n* = 36,196)	(*n* = 24,474)		
The JDI8 score	0–2	3	4, 5	6–8		
Person-years	285,843	267,100	636,870	445,489		
All-cause mortality						
No. of death	3159	3381	8323	5733		
Model 1^†^	1.00 (ref)	0.93 (0.89–0.98)	0.86 (0.82–0.89)	0.77 (0.74–0.81)		< 0.001
Model 2^‡^	1.00 (ref)	0.94 (0.90–0.99)	0.90 (0.86–0.94)	0.84 (0.80–0.88)		< 0.001
Model 3^§^	1.00 (ref)	0.95 (0.90–0.99)	0.91 (0.87–0.95)	0.86 (0.81–0.90)		< 0.001
Model 3^§^					0.97 (0.96–0.98)	< 0.001
Cancer mortality						
No. of death	1059	1129	2883	2077		
Model 1^†^	1.00 (ref)	0.97 (0.89–1.06)	0.95 (0.88–1.02)	0.89 (0.82–0.96)		0.003
Model 2^‡^	1.00 (ref)	0.98 (0.90–1.07)	0.98 (0.91–1.06)	0.94 (0.87–1.02)		0.140
Model 3^§^	1.00 (ref)	0.99 (0.91–1.07)	0.98 (0.91–1.06)	0.94 (0.86–1.03)		0.175
Model 3^§^					0.99 (0.97–1.00)	0.144
Cardiovascular disease mortality						
No. of death	727	858	2006	1399		
Model 1^†^	1.00 (ref)	0.99 (0.89–1.09)	0.85 (0.77–0.92)	0.76 (0.69–0.84)		< 0.001
Model 2^‡^	1.00 (ref)	1.00 (0.90–1.10)	0.90 (0.82–0.99)	0.86 (0.78–0.95)		< 0.001
Model 3^§^	1.00 (ref)	1.01 (0.91–1.11)	0.92 (0.84–1.01)	0.89 (0.80–0.99)		0.007
Model 3^§^					0.97 (0.95–0.99)	0.003
Heart disease mortality						
No. of death	381	470	1,051	698		
Model 1^†^	1.00 (ref)	1.04 (0.91–1.20)	0.87 (0.77–0.99)	0.77 (0.67–0.88)		< 0.001
Model 2^‡^	1.00 (ref)	1.05 (0.92–1.21)	0.93 (0.82–1.05)	0.87 (0.75–0.99)		0.005
Model 3^§^	1.00 (ref)	1.06 (0.93–1.22)	0.95 (0.84–1.07)	0.89 (0.77–1.03)		0.037
Model 3^§^					0.96 (0.94–0.99)	0.008
Cerebrovascular disease mortality						
No. of death	283	298	784	585		
Model 1^†^	1.00 (ref)	0.86 (0.73–1.01)	0.81 (0.70–0.93)	0.76 (0.65–0.89)		0.001
Model 2^‡^	1.00 (ref)	0.87 (0.74–1.02)	0.86 (0.74–0.99)	0.85 (0.72–0.99)		0.061
Model 3^§^	1.00 (ref)	0.88 (0.74–1.03)	0.88 (0.76–1.02)	0.89 (0.75–1.05)		0.237
Model 3^§^					0.98 (0.95–1.01)	0.275

Adjusted hazard ratios (HR) and 95% confidence intervals. Analysis by Cox proportional hazards model

^†^Model 1 was adjusted for age (45–49, 50–54, 55–59, 60–64, 65–69 or ≥ 70 years), sex, and study area (11 areas)

^‡^Model 2 was adjusted as for model 1 plus BMI (< 18.5, 18.5–24.9, 25–29.9, 30 ≥ kg/m^2^, or missing), smoking status (current, former, never, or missing), alcohol drinking (< 1 time/month, 1–3 times/month, 1–2, 3–4, 5–6 times/week, every day, or missing), total physical activity (quartile of metabolic equivalent task-hours/day), medication (antihypertensive, cholesterol-lowering, or hypoglycemic agents [yes or no for each item]), and occupation (agriculture, forestry, fishery, salaried, self-employed, specialty work, housework, unemployed, or other [yes or no for each item])

^§^Model 3 was adjusted as for model 2 plus total energy intake (in kcal/d; sex-specific quartile categories)

These results did not change substantially after excluding 1,380 participants who died in the first 3 years of follow up (Supplemental Table 2). In addition, these inverse associations did not differ between the sexes (Supplemental Table 3). To minimize potential bias attributed to total energy intake, even when we used the data after adjustment by the residual method (Supplemental Table 4) and also when including participants who reported extreme total energy intake (Supplemental Table 5), the JDI8 score was inversely associated with the risk of all-cause, CVD, and heart disease mortality.

Table 3 shows the associations between each component of the JDI8 and all-cause, cancer, CVD, heart disease, and cerebrovascular disease mortality. Except for rice and miso soup, consuming the five other adherence components resulted in a significant inverse association with all-cause mortality; the multivariable-adjusted HRs (95% CI) were 0.94 (0.92–0.97) for seaweeds, 0.95 (0.92–0.98) for pickles, 0.94 (0.91–0.96) for green and yellow vegetables, 0.97 (0.94–0.997) for fish, and 0.89 (0.86–0.91) for green tea. No significant association was observed between the non-adherence component and all-cause mortality. The multivariable-adjusted HR (95% CI) was 1.02 (0.99–1.05) for beef and pork.

**Table 3:** Relationship between the JDI8 score components and mortality (*n* = 92,969)

	All-cause mortality		Cancer mortality		Cardiovascular disease mortality		Heart disease mortality		Cerebrovascular disease mortality	
	0 point	One point	0 point	One point	0 point	One point	0 point	One point	0 point	One point
Adhering components										
Rice^†^	1.00 (ref)	1.02 (0.99–1.06)	1.00 (ref)	0.98 (0.93–1.03)	1.00 (ref)	1.07 (1.00–1.14)	1.00 (ref)	1.09 (1.00–1.19)	1.00 (ref)	1.09 (0.99–1.21)
Miso soup^†^	1.00 (ref)	0.98 (0.95–1.01)	1.00 (ref)	1.02 (0.97–1.08)	1.00 (ref)	0.99 (0.93–1.06)	1.00 (ref)	1.03 (0.94–1.12)	1.00 (ref)	0.97 (0.88–1.08)
Seaweeds^†^	1.00 (ref)	0.94 (0.92–0.97)	1.00 (ref)	0.97 (0.93–1.02)	1.00 (ref)	0.93 (0.88–0.99)	1.00 (ref)	0.94 (0.87–1.02)	1.00 (ref)	0.92 (0.84–1.01)
Pickles^†^	1.00 (ref)	0.95 (0.92–0.98)	1.00 (ref)	1.03 (0.97–1.08)	1.00 (ref)	0.96 (0.90–1.02)	1.00 (ref)	0.93 (0.85–1.02)	1.00 (ref)	0.997 (0.90–1.11)
Green and yellow vegetables^†^	1.00 (ref)	0.94 (0.91–0.96)	1.00 (ref)	0.96 (0.92–1.02)	1.00 (ref)	0.92 (0.87–0.98)	1.00 (ref)	0.89 (0.82–0.97)	1.00 (ref)	0.97 (0.88–1.07)
Fish^†^	1.00 (ref)	0.97 (0.94–0.997)	1.00 (ref)	0.98 (0.93–1.03)	1.00 (ref)	0.98 (0.92–1.04)	1.00 (ref)	1.00 (0.92–1.10)	1.00 (ref)	0.91 (0.82–1.01)
Green tea^†^	1.00 (ref)	0.89 (0.86–0.91)	1.00 (ref)	0.96 (0.91–1.00)	1.00 (ref)	0.88 (0.83–0.93)	1.00 (ref)	0.81 (0.75–0.88)	1.00 (ref)	0.95 (0.86–1.04)
Non-adhering components										
Beef and pork^†^	1.00 (ref)	1.02 (0.99–1.05)	1.00 (ref)	1.00 (0.95–1.05)	1.00 (ref)	1.01 (0.95–1.08)	1.00 (ref)	0.98 (0.90–1.07)	1.00 (ref)	1.03 (0.93–1.14)

Adjusted hazard ratios (HR) and 95% confidence intervals. Analysis by Cox proportional hazards model

^†^Model was adjusted for age (45–49, 50–54, 55–59, 60–64, 65–69 or ≥ 70 years), sex, study area (11 areas), BMI (< 18.5, 18.5–24.9, 25–29.9, 30 ≥ kg/m^2^, or missing), smoking status (current, former, never, or missing), alcohol drinking (< 1 time/month, 1–3 times/month, 1–2, 3–4, 5–6 times/week, every day, or missing), total physical activity (quartile of metabolic equivalent task-h/day), medication (antihypertensive, cholesterol-lowering, or hypoglycemic agents [yes or no for each item]), occupation (agriculture, forestry, fishery, office work, self-employed, specialty work, housewife, unemployed, or other [yes or no for each item]), and total energy intake (in kcal/day; sex-specific quartile categories)

In addition, when we performed the analysis that included sodium and soybean products intake in the covariates, we observed an inverse association between the JDI8 score and the risk of all-cause, CVD, and heart disease mortality as well as cancer mortality risk (Supplemental Table 6).

## Discussion

We investigated the association between adherence to the Japanese diet and all-cause and cause-specific mortality in this cohort study covering multiple areas of Japan. We observed that a higher JDI8 score was significantly associated with lower risks of all-cause, CVD, and heart disease mortality.

Our findings of inverse associations between adherence to the Japanese diet and all-cause and CVD mortality are consistent with previous studies, which reported that both sexes who adhered to the Japanese diet had a 9% lower risk of all-cause mortality [2] and a 26% lower risk of CVD mortality [3]. Therefore, the results of our large-scale cohort from settings all over Japan support the findings in these previous studies.

In our analysis using each component of the JDI8 as an exposure variable, we found that higher consumption of seaweeds, pickles, green and yellow vegetables, fish, and green tea were associated with a decreased risk of all-cause, CVD, and heart disease mortality. These foods contain many beneficial nutrients, for instance, vegetables contains a myriad of nutrients and phytochemicals, including fiber, vitamin C, potassium, carotenoids, antioxidants, and flavonoids [15]; fish contains very-long-chain fatty acids, EPA, and DHA [16]; green tea contains polyphenol catechins with antioxidative activity [17, 18]; and seaweeds contains dietary fiber [19].

Although the impact of individual components of the JDI8 score upon mortality risk was small, the impact of the JDI8 score as a whole (i.e., the Japanese dietary pattern) was stronger. This finding may be explained by the cumulative effects of individual components of the Japanese diet. A previous study suggested that owing to the complex biological interactions between different components of the diet, the use of a whole diet approach rather than individual nutrients or food groups might help to understand the role of diet in health outcomes [20].

In this study, participants with higher JDI8 score consumed sodium, which increases the risk of gastric cancer [21, 22] and hypertension [23, 24], and rice that raises glycemic indices, which increases the risk of colorectal cancer [21] and cardiometabolic complications [25]. On the other hand, participants with higher JDI8 score may also consume such beneficial nutrients as protein, fiber, vitamins A, C, and E, calcium, iron, potassium, and magnesium which have been associated with lower risk for CVD, including heart disease and stroke [26–34], because their intake of all adhering components of JDI8 are high.

Additionally, the present study indicated that intake of potassium as well as sodium increased among the subjects with higher JDI8 score, and the sodium–potassium ratio was almost the same among all the groups. It was reported that sodium–potassium ratio predicted the risk for cardiovascular events better than sodium intake alone [35]. Evidence also indicates that high potassium intake can mitigate the risk for high blood pressure despite a high-sodium diet [36, 37]. Therefore, the inverse association of the Japanese diet assessed by the JDI8 with mortality may be partly explained by the combined effect of these nutrients.

On the other hand, we did not observe a significant association between the JDI8 score and cancer mortality. According to the World Cancer Research Fund [21] and the National Cancer Center of Japan [22], vegetable intake was associated with a lower risk for stomach and esophagus cancer; fish intake was associated with a lower risk for cervical cancer; pickles intake was associated with a higher risk for stomach cancer; red meat and processed meat intake were associated with a higher risk for colorectal cancer; and dietary fiber and calcium intake were associated with a lower risk for colorectal cancer. These findings of cancer incidence imply that the association between foods or nutrients and cancer differ by site of cancer. However, less is known about the association between diet and cancer mortality. Other factors, such as stage at diagnosis or treatment, are important contributors to cancer mortality outcomes and were not adjusted for in these analyses. Thus, further investigation of the association between the Japanese diet and cause-specific cancer is required.

Our study had several strengths: (1) it was a large population-based cohort study including 92,969 persons; (2) the response rate was relatively high (79.2%); (3) the follow-up period was long enough for us to eliminate possible biases by conducting analysis excluding participants who died in the first 3 years of follow-up; (4) few participants were lost during follow-up (0.3%) and an almost complete follow-up for cause of death (in decedents) was possible among all subjects (over 98%); (5) we used the validated semi-quantitative FFQ; and (6) many confounding factors were taken into account.

However, there were also several limitations. First, because dietary intake was assessed at only one time point, changes in dietary habits were not considered. Second, although we adjusted for many potential confounders, residual confounding such as socioeconomic status [38–40] could not be completely ruled out.

In conclusion, the present study indicated that the Japanese diet assessed by the JDI8 is associated with a decreased risk of all-cause, CVD, and heart disease mortality among Japanese.

## Electronic supplementary material

Below is the link to the electronic supplementary material.

Supplementary file1 (DOCX 98 kb)

## Abbreviations

CVD: Cardiovascular disease
JDI8: 8-Item Japanese Diet Index
JDI: Japanese Diet Index
JPHC: Japan Public Health Center-based Prospective Study
FFQ: Food frequency questionnaire
ICD-10: International Classification of Diseases, 10th revision
HR: Hazard ratio
CI: Confidence interval
SD: Standard deviation
