# Supplementary material for: Association between adherence to the Japanese diet and all-cause and cause-specific mortality: the Japan Public Health Center-based Prospective Study
Source: Eur J Nutr. 2020 Jul 16;60(3):1327–36. doi: 10.1007/s00394-020-02330-0 (PMC7987617; doi:10.1007/s00394-020-02330-0)
Supplement: Supplementary file 1 — Supplementary file1 (DOCX 98 kb) [file 394_2020_2330_MOESM1_ESM.docx]

**Supplemental Table 1.** The sex-specific median of intake of the JDI8 components (male = 42,700, female = 50,269).

|  |  | The sex-specific median (g/day) | |  |
| --- | --- | --- | --- | --- |
|  |  | Male | Female |  |
|  | Rice | 420.0 | 330.0 |  |
|  | Miso soup | 225.0 | 150.0 |  |
|  | Seaweeds | 6.6 | 9.0 |  |
|  | Pickles | 18.9 | 21.6 |  |
|  | Green and yellow vegetables | 47.3 | 66.4 |  |
|  | Fish | 76.7 | 74.9 |  |
|  | Green tea | 300.0 | 394.3 |  |
|  | Beef and pork | 41.2 | 36.5 |  |

**Supplemental Table 2.** Relationship between the JDI8 score and mortality after exclusion of deaths occurring in the first 3 years of follow-up (n = 91,589)*. (Adjusted hazard ratios (HR) and 95% confidence intervals)

| 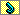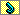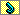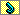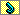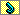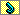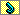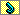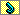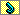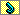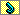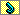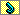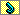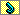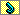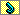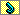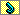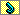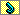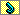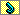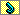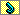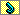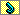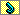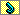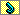 | Groups of the JDI8 score | | | |  |
| --- | --- | --- | --- | --- | --- |
|  | G1 (low) | G2 | G3 | G4 (high) |  |
|  | (n = 16,585) | (n = 15,244) | (n = 35,627) | (n = 24,133) | *P*-trend |
| The JDI8 score | 0–2 | 3 | 4, 5 | 6–8 |  |
| Person-years | 285,425 | 266,731 | 635,935 | 444,919 |  |
| **All-cause mortality** |  |  |  |  |  |
| No. of death | 2,906 | 3,164 | 7,754 | 5,392 |  |
| Model 1^†^ | 1.00 (ref) | 0.94 (0.90–0.99) | 0.86 (0.83–0.90) | 0.78 (0.74–0.82) | <0.001 |
| Model 2^‡^ | 1.00 (ref) | 0.96 (0.91–1.00) | 0.91 (0.87–0.95) | 0.85 (0.81–0.89) | <0.001 |
| Model 3^§^ | 1.00 (ref) | 0.96 (0.91–1.01) | 0.91 (0.87–0.96) | 0.86 (0.82–0.91) | <0.001 |
| **Cancer mortality** |  |  |  |  |  |
| No. of death | 967 | 1,039 | 2,656 | 1,907 |  |
| Model 1^†^ | 1.00 (ref) | 0.98 (0.90–1.07) | 0.96 (0.88–1.03) | 0.89 (0.82–0.97) | 0.003 |
| Model 2^‡^ | 1.00 (ref) | 0.99 (0.91–1.09) | 0.99 (0.92–1.07) | 0.94 (0.86–1.02) | 0.132 |
| Model 3^§^ | 1.00 (ref) | 1.00 (0.91–1.09) | 0.99 (0.92–1.07) | 0.94 (0.86–1.03) | 0.150 |
| **Cardiovascular disease mortality** |  |  |  |  |  |
| No. of death | 664 | 801 | 1,858 | 1,320 |  |
| Model 1^†^ | 1.00 (ref) | 1.00 (0.90–1.11) | 0.85 (0.78–0.93) | 0.78 (0.71–0.86) | <0.001 |
| Model 2^‡^ | 1.00 (ref) | 1.02 (0.92–1.13) | 0.90 (0.82–0.99) | 0.87 (0.78–0.96) | 0.001 |
| Model 3^§^ | 1.00 (ref) | 1.02 (0.92–1.13) | 0.92 (0.84–1.01) | 0.90 (0.80–0.999) | 0.010 |
| **Heart disease mortality** |  |  |  |  |  |
| No. of death | 345 | 442 | 974 | 654 |  |
| Model 1^†^ | 1.00 (ref) | 1.08 (0.94–1.24) | 0.89 (0.78–1.01) | 0.78 (0.68–0.90) | <0.001 |
| Model 2^‡^ | 1.00 (ref) | 1.09 (0.94–1.26) | 0.94 (0.83–1.07) | 0.88 (0.76–1.01) | 0.007 |
| Model 3^§^ | 1.00 (ref) | 1.09 (0.95–1.26) | 0.95 (0.83–1.09) | 0.89 (0.77–1.04) | 0.031 |
| **Cerebrovascular disease mortality** |  |  |  |  |  |
| No. of death | 261 | 272 | 720 | 554 |  |
| Model 1^†^ | 1.00 (ref) | 0.84 (0.71–1.00) | 0.80 (0.69–0.92) | 0.77 (0.66–0.90) | 0.002 |
| Model 2^‡^ | 1.00 (ref) | 0.85 (0.72–1.01) | 0.84 (0.73–0.98) | 0.85 (0.72–0.998) | 0.094 |
| Model 3^§^ | 1.00 (ref) | 0.86 (0.73–1.02) | 0.87 (0.75–1.01) | 0.90 (0.75–1.06) | 0.334 |
| *Analysis by Cox proportional hazards model. | | | | | |
| ^†^Model 1 was adjusted for age (45–49, 50–54, 55–59, 60–64, 65–69 or ≥70 years), sex, and study area (11 areas). | | | | | |
| ^‡^Model 2 was adjusted as for model 1 plus BMI (<18.5, 18.5–24.9, 25–29.9, 30≥ kg/m^2^, or missing), smoking status (current, former, never, or missing), alcohol drinking (<1 time/month, 1–3 times/month, 1–2, 3–4, 5–6 times/week, every day, or missing), total physical activity (quartile of metabolic equivalent task-hours/day), medication (antihypertensive, cholesterol-lowering or hypoglycemic agents [yes or no for each item]), and occupation (agriculture, forestry, fishery, office work, self-employed, specialty work, housewife, unemployed, or other [yes or no for each item]). | | | | | |
| ^§^Model 3 was adjusted as for model 2 plus total energy intake (in kcal/d; sex-specific quartile categories). | | | | | |

**Supplemental Table 3.** Relationship between the JDI8 score and mortality by sex (male = 42,700, female = 50,269)*.

| 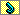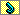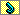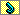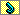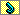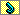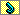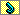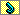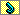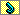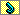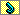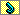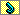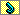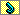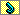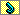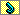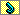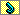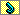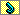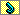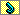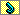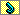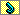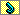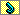 | Groups of the JDI8 score | | | |  |  |
| --- | --- | --- | --- | --- | --- | --- |
|  | G1 (low) | G2 | G3 | G4 (high) | *P*-trend | *P*-interaction |
| **All-cause mortality** |  |  |  |  |  |  |
| Male^†^ | 1.00 (ref) | 0.96 (0.90–1.03) | 0.93 (0.88–0.99) | 0.90 (0.84–0.96) | 0.001 | 0.211 |
| Female^†^ | 1.00 (ref) | 0.92 (0.86–0.996) | 0.89 (0.84–0.96) | 0.81 (0.75–0.88) | <0.001 |  |
| **Cancer mortality** |  |  |  |  |  |  |
| Male^†^ | 1.00 (ref) | 0.97 (0.87–1.08) | 0.95 (0.86–1.05) | 0.93 (0.83–1.04) | 0.188 | 0.611 |
| Female^†^ | 1.00 (ref) | 1.01 (0.88–1.16) | 1.03 (0.91–1.16) | 0.95 (0.82–1.10) | 0.578 |  |
| **Cardiovascular disease mortality** |  |  |  |  |  |  |
| Male^†^ | 1.00 (ref) | 0.97 (0.85–1.12) | 0.94 (0.83–1.06) | 0.92 (0.80–1.06) | 0.222 | 0.356 |
| Female^†^ | 1.00 (ref) | 1.06 (0.91–1.22) | 0.92 (0.80–1.05) | 0.86 (0.74–1.02) | 0.020 |  |
| **Heart disease mortality** |  |  |  |  |  |  |
| Male^†^ | 1.00 (ref) | 1.03 (0.85–1.24) | 0.96 (0.82–1.14) | 0.91 (0.76–1.11) | 0.235 | 0.442 |
| Female^†^ | 1.00 (ref) | 1.12 (0.91–1.37) | 0.95 (0.79–1.15) | 0.90 (0.71–1.12) | 0.148 |  |
| **Cerebrovascular disease mortality** |  |  |  |  |  |  |
| Male^†^ | 1.00 (ref) | 0.89 (0.71–1.11) | 0.93 (0.76–1.14) | 0.95 (0.76–1.19) | 0.900 | 0.652 |
| Female^†^ | 1.00 (ref) | 0.87 (0.68–1.11) | 0.83 (0.67–1.03) | 0.82 (0.64–1.06) | 0.138 |  |
| *Analysis by Cox proportional hazards model. | | | | | | |
| ^†^Model was adjusted for age (45–49, 50–54, 55–59, 60–64, 65–69 or ≥70 years), study area (11 areas), BMI (<18.5, 18.5–24.9, 25–29.9, 30≥ kg/m^2^, or missing), smoking status (current, former, never, or missing), alcohol drinking (<1 time/month, 1–3 times/month, 1–2, 3–4, 5–6 times/week, every day, or missing), total physical activity (quartile of metabolic equivalent task-hours/day), medication (antihypertensive, cholesterol-lowering or hypoglycemic agents [yes or no for each item]), occupation (agriculture, forestry, fishery, office work, self-employed, specialty work, housewife, unemployed, or other [yes or no for each item]), and total energy intake (in kcal/d; sex-specific quartile categories). | | | | | | |

**Supplemental Table 4.** Relationship between the JDI8 score by the residual method and mortality (n = 92,969)*. (Adjusted hazard ratios (HR) and 95% confidence intervals)

| 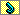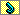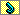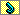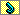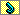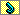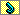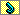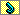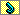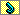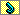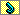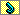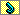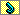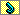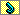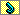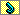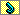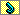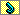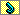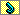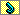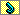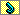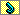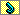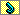 | Groups of the JDI8 score | | | |  |
| --- | --- | --- | --- | --- | --- |
|  | G1 (low) | G2 | G3 | G4 (high) |  |
|  | (n = 20,023) | (n = 18,200) | (n = 20,211) | (n = 34,535) | *P*-trend |
| The JDI8 score | 0–2 | 3 | 4 | 5–8 |  |
| Person-years | 342,743 | 318,240 | 355,571 | 618,748 |  |
| **All-cause mortality** |  |  |  |  |  |
| No. of death | 3,633 | 3,783 | 4,669 | 8,511 |  |
| Model 1^†^ | 1.00 (ref) | 0.92 (0.88–0.96) | 0.90 (0.86–0.95) | 0.85 (0.81–0.88) | <0.001 |
| Model 2^‡^ | 1.00 (ref) | 0.93 (0.89–0.97) | 0.93 (0.89–0.97) | 0.89 (0.85–0.93) | <0.001 |
| **Cancer mortality** |  |  |  |  |  |
| No. of death | 1,272 | 1,271 | 1,643 | 2,962 |  |
| Model 1^†^ | 1.00 (ref) | 0.92 (0.85–1.00) | 0.97 (0.90–1.05) | 0.91 (0.85–0.98) | 0.046 |
| Model 2^‡^ | 1.00 (ref) | 0.94 (0.87–1.02) | 1.00 (0.93–1.08) | 0.97 (0.90–1.04) | 0.640 |
| **Cardiovascular disease mortality** |  |  |  |  |  |
| No. of death | 844 | 940 | 1,077 | 2,129 |  |
| Model 1^†^ | 1.00 (ref) | 0.94 (0.86–1.03) | 0.85 (0.77–0.93) | 0.85 (0.78–0.93) | <0.001 |
| Model 2^‡^ | 1.00 (ref) | 0.95 (0.87–1.05) | 0.87 (0.79–0.95) | 0.89 (0.82–0.97) | 0.007 |
| **Heart disease mortality** |  |  |  |  |  |
| No. of death | 444 | 500 | 567 | 1,089 |  |
| Model 1^†^ | 1.00 (ref) | 0.96 (0.85–1.10) | 0.87 (0.76–0.99) | 0.86 (0.77–0.97) | 0.007 |
| Model 2^‡^ | 1.00 (ref) | 0.97 (0.85–1.10) | 0.88 (0.78–1.00) | 0.90 (0.80–1.01) | 0.046 |
| **Cerebrovascular disease mortality** |  |  |  |  |  |
| No. of death | 322 | 355 | 422 | 851 |  |
| Model 1^†^ | 1.00 (ref) | 0.91 (0.78–1.06) | 0.83 (0.72–0.97) | 0.83 (0.73–0.96) | 0.010 |
| Model 2^‡^ | 1.00 (ref) | 0.93 (0.80–1.08) | 0.87 (0.74–1.01) | 0.89 (0.78–1.03) | 0.124 |
| *Analysis by Cox proportional hazards model. | | | | | |
| ^†^Model 1 was adjusted for age (45–49, 50–54, 55–59, 60–64, 65–69 or ≥70 years), sex, and study area (11 areas). | | | | | |
| ^‡^Model 2 was adjusted as for model 1 plus BMI (<18.5, 18.5–24.9, 25–29.9, 30≥ kg/m^2^, or missing), smoking status (current, former, never, or missing), alcohol drinking (<1 time/month, 1–3 times/month, 1–2, 3–4, 5–6 times/week, every day, or missing), total physical activity (quartile of metabolic equivalent task-hours/day), medication (antihypertensive, cholesterol-lowering, or hypoglycemic agents [yes or no for each item]), and occupation (agriculture, forestry, fishery, office work, self-employed, specialty work, housewife, unemployed, or other [yes or no for each item]). | | | | | |

**Supplemental Table 5.** Relationship between the JDI8 score and mortality including participants who reported extreme total energy intake (n = 97,802)*. (Adjusted hazard ratios (HR) and 95% confidence intervals)

| 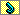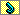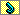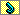 | Groups of the JDI8 score | | | |  |
| --- | --- | --- | --- | --- | --- |
|  | G1 (low) | G2 | G3 | G4 (high) |  |
|  | (n = 18,303) | (n = 16,122) | (n = 37,359) | (n = 26,018) | *P*-trend |
| The JDI8 score | 0–2 | 3 | 4, 5 | 6–8 |  |
| Person-years | 309,657 | 277,562 | 656,604 | 473,553 |  |
| **All-cause mortality** |  |  |  |  |  |
| No. of death | 3,726 | 3,630 | 8,646 | 6,092 |  |
| Model 1^†^ | 1.00 (ref) | 0.91 (0.87–0.95) | 0.83 (0.80–0.87) | 0.75 (0.72–0.78) | <0.001 |
| Model 2^‡^ | 1.00 (ref) | 0.93 (0.89–0.97) | 0.89 (0.85–0.92) | 0.83 (0.79–0.87) | <0.001 |
| Model 3^§^ | 1.00 (ref) | 0.94 (0.89–0.98) | 0.90 (0.87–0.94) | 0.85 (0.81–0.89) | <0.001 |
| **Cancer mortality** |  |  |  |  |  |
| No. of death | 1,205 | 1,197 | 2,981 | 2,219 |  |
| Model 1^†^ | 1.00 (ref) | 0.97 (0.89–1.05) | 0.94 (0.88–1.01) | 0.89 (0.82–0.96) | 0.001 |
| Model 2^‡^ | 1.00 (ref) | 0.98 (0.91–1.07) | 0.98 (0.91–1.05) | 0.95 (0.88–1.02) | 0.178 |
| Model 3^§^ | 1.00 (ref) | 0.98 (0.91–1.07) | 0.98 (0.91–1.06) | 0.95 (0.87–1.03) | 0.200 |
| **Cardiovascular disease mortality** |  |  |  |  |  |
| No. of death | 887 | 923 | 2,091 | 1,474 |  |
| Model 1^†^ | 1.00 (ref) | 0.94 (0.86–1.03) | 0.80 (0.74–0.87) | 0.71 (0.65–0.78) | <0.001 |
| Model 2^‡^ | 1.00 (ref) | 0.96 (0.87–1.05) | 0.87 (0.80–0.94) | 0.81 (0.74–0.89) | <0.001 |
| Model 3^§^ | 1.00 (ref) | 0.97 (0.89–1.07) | 0.90 (0.83–0.98) | 0.86 (0.78–0.95) | 0.001 |
| **Heart disease mortality** |  |  |  |  |  |
| No. of death | 484 | 505 | 1,098 | 736 |  |
| Model 1^†^ | 1.00 (ref) | 0.95 (0.84–1.08) | 0.80 (0.71–0.89) | 0.69 (0.61–0.78) | <0.001 |
| Model 2^‡^ | 1.00 (ref) | 0.97 (0.86–1.10) | 0.87 (0.77–0.97) | 0.79 (0.70–0.90) | <0.001 |
| Model 3^§^ | 1.00 (ref) | 0.99 (0.87–1.12) | 0.90 (0.80–1.01) | 0.84 (0.73–0.96) | 0.005 |
| **Cerebrovascular disease mortality** |  |  |  |  |  |
| No. of death | 337 | 318 | 817 | 620 |  |
| Model 1^†^ | 1.00 (ref) | 0.83 (0.71–0.97) | 0.79 (0.69–0.90) | 0.73 (0.63–0.84) | <0.001 |
| Model 2^‡^ | 1.00 (ref) | 0.85 (0.73–0.99) | 0.85 (0.74–0.97) | 0.82 (0.71–0.95) | 0.020 |
| Model 3^§^ | 1.00 (ref) | 0.86 (0.74–1.01) | 0.88 (0.77–1.01) | 0.88 (0.75–1.03) | 0.210 |
| *Analysis by Cox proportional hazards model. | | | | | |
| ^†^Model 1 was adjusted for age (45–49, 50–54, 55–59, 60–64, 65–69 or ≥70 years), sex, and study area (11 areas). | | | | | |
| ^‡^Model 2 was adjusted as for model 1 plus BMI (<18.5, 18.5–24.9, 25–29.9, 30≥ kg/m^2^, or missing), smoking status (current, former, never, or missing), alcohol drinking (<1 time/month, 1–3 times/month, 1–2, 3–4, 5–6 times/week, every day, or missing), total physical activity (quartile of metabolic equivalent task-hours/day), medication (antihypertensive, cholesterol-lowering, or hypoglycemic agents [yes or no for each item]), and occupation (agriculture, forestry, fishery, office work, self-employed, specialty work, housewife, unemployed, or other [yes or no for each item]). | | | | | |
| ^§^Model 3 was adjusted as for model 2 plus total energy intake (in kcal/d; sex-specific quartile categories). | | | | | |

**Supplemental Table 6.** Relationship between the JDI8 score and mortality (n = 92,969)*. (Adjusted hazard ratios (HR) and 95% confidence intervals)

|  | Groups of the JDI8 score | | | |  |
| --- | --- | --- | --- | --- | --- |
|  | G1 (low) | G2 | G3 | G4 (high) |  |
|  | (n = 16,838) | (n = 15,461) | (n = 36,196) | (n = 24,474) | *P*-trend |
| The JDI8 score | 0–2 | 3 | 4, 5 | 6–8 |  |
| Person-years | 285,843 | 267,100 | 636,870 | 445,489 |  |
| **All-cause mortality** |  |  |  |  |  |
| No. of death | 3,159 | 3,381 | 8,323 | 5,733 |  |
| Model A^†^ | 1.00 (ref) | 0.94 (0.89–0.99) | 0.90 (0.86–0.94) | 0.84 (0.79–0.88) | <0.001 |
| Model B^‡^ | 1.00 (ref) | 0.94 (0.89–0.99) | 0.90 (0.86–0.94) | 0.84 (0.79–0.89) | <0.001 |
| **Cancer mortality** |  |  |  |  |  |
| No. of death | 1,059 | 1,129 | 2,883 | 2,077 |  |
| Model A^†^ | 1.00 (ref) | 0.97 (0.89–1.06) | 0.95 (0.88–1.03) | 0.90 (0.82–0.99) | 0.023 |
| Model B^‡^ | 1.00 (ref) | 0.96 (0.89–1.05) | 0.94 (0.87–1.02) | 0.89 (0.81–0.98) | 0.014 |
| **Cardiovascular disease mortality** |  |  |  |  |  |
| No. of death | 727 | 858 | 2,006 | 1,399 |  |
| Model A^†^ | 1.00 (ref) | 0.995 (0.90–1.10) | 0.90 (0.82–0.99) | 0.86 (0.77–0.96) | 0.002 |
| Model B^‡^ | 1.00 (ref) | 1.00 (0.90–1.11) | 0.91 (0.83–1.00) | 0.87 (0.78–0.98) | 0.005 |
| **Heart disease mortality** |  |  |  |  |  |
| No. of death | 381 | 470 | 1,051 | 698 |  |
| Model A^†^ | 1.00 (ref) | 1.06 (0.92–1.21) | 0.94 (0.82–1.07) | 0.89 (0.76–1.03) | 0.043 |
| Model B^‡^ | 1.00 (ref) | 1.06 (0.92–1.22) | 0.95 (0.83–1.08) | 0.90 (0.77–1.05) | 0.063 |
| **Cerebrovascular disease mortality** |  |  |  |  |  |
| No. of death | 283 | 298 | 784 | 585 |  |
| Model A^†^ | 1.00 (ref) | 0.87 (0.74–1.02) | 0.86 (0.74–1.00) | 0.85 (0.71–1.02) | 0.113 |
| Model B^‡^ | 1.00 (ref) | 0.87 (0.74–1.03) | 0.87 (0.75–1.01) | 0.86 (0.72–1.03) | 0.147 |
| *Analysis by Cox proportional hazards model. | | | | | |
| ^†^Model A was adjusted as for Model 3 at Table 2 plus sodium intake (in mg/d; sex-specific quartile categories). | | | | | |
| ^‡^Model B was adjusted as for model A plus soybean products intake (in g/d; sex-specific quartile categories). | | | | | |
